# Editorial: Chronic rheumatic inflammatory conditions and cardiovascular health

**DOI:** 10.3389/fmed.2022.953756

**Published:** 2022-08-16

**Authors:** Alberto Lo Gullo, Giuseppe Mandraffino

**Affiliations:** ^1^Unit of Rheumatology, Department of Medicine, ARNAS Garibaldi Hospital, Catania, Italy; ^2^Internal Medicine Unit, Department of Clinical and Experimental Medicine, Lipid Center, University of Messina, Messina, Italy

**Keywords:** rheumatic, inflammatory conditions and cardiovascular health, rheumatic and musculoskeletal disease, cardiovascular diseases, vasculitis, Pulse wave velocity (PWV), arterial stiffness

Patients with chronic inflammatory rheumatic diseases (IRD), such as systemic lupus erythematosus (SLE), chronic arthritis and vasculitis, have a higher risk of developing premature cardiovascular disease (CVD) (1, 2). The multifactorial characteristic of this condition is thought to result from an interaction of inflammation, metabolic factors, therapy- and disease-related factor. Standardized mortality ratios in patients with IRD are higher than those in the general population (3); this increased and commonly premature mortality is mainly due to cardiovascular events (CV). Interestingly, this increased risk of CVD in rheumatoid arthritis (RA) is comparable to that observed in type 2 diabetes mellitus (4). Dampening disease activity has been associated with reduction in the CV mortality of patients with IRD; furthermore treatment with anti TNF or DMARDs seem to reduce CV events (5). European League Against Rheumatism's (EULAR) evidence-based recommendations for CV risk management in patients with RA and other forms of IRD strongly support the use of algorithms to stratify the CV risk of patients with inflammatory arthritis. Briefly, to avoid the underestimation of CV risk, the EULAR task force recommends a 1.5 multiplication factor in patients with RA who meet two of the three following criteria: disease duration longer than 10 years, presence of rheumatoid factor or anti-CCP antibodies, and extra-articular manifestations (6). Taking together these observations, with this special issue the impact on cardiovascular health was assessed in the management of patients with several rheumatic conditions including chronic arthritis, connective tissue disease and vasculitis.

A systematic review on arterial stiffness in vasculitis was conducted, particularly focusing on ANCA vasculitis, Behcet disease and Takayasu Arteritis; vascular properties impairment, as measured by Pulse wave velocity, is reported in most subtypes of vasculitis, underlining the importance of an effective—and early—treatment of conventional CV risk factors, and calling for additional investigation on further ways to mitigate the risk excess (Lo Gullo et al.).

Skoog et al. reported that the examination—through an extended ultrasound protocol—of brachiocephalic and common carotid arteries can provide a very high diagnostic sensitivity in patients with suspected giant cell arteritis without affecting the specificity when temporal and axillary findings are indecisive. Kuret et al. investigated some markers potentially helpful in predicting giant cell arteritis relapses suggesting that IL-23 might be a promising biomarker of uncontrolled and active disease and could give early indication of upcoming relapses.

An update about cardiovascular risk in arthritis was also provided. Argnani et al. in a big Italian study reported that RA patients had higher incidence of atrial fibrillation (incidence rate ratio, IRR 1.28), heart failure (IRR 1.53), stroke (IRR 1.19), and myocardial infarction compared to controls. On the other hand, as reported by Schirmer, patients with spondyloarthritis, psoriatic arthritis and rheumatoid arthritis have a prevalence of CVD that is 8.7, 12.8, and 18.7% respectively (Yagensky and Schirmer). Degboè et al. reported a higher prevalence of cardiovascular risk factors (CVRs), as well as a higher prevalence of major adverse cardiovascular events in PsA compared to the general population. The CVR is higher in the PsA population than in the controls either using SCORE and QRISK2 equations or using SCORE- PsA and QRISK2-PsA equations. Hupin et al. investigated the autonomic function in RA, and in particular the parasympathetic tone, using the heart rate recovery (HRR), which improved after 1 and 2 year by guided physical activity. As regards physical activity, Garcia et al. describes the beneficial effect of personalized training program to improve aerobic activity and glycated hemoglobin in Sjogren Syndrome. Svensson et al. found impaired microcirculation in systemic lupus erythematosus as reflected by peak oxygen saturation even in the younger ages, and higher Augmentation index as compared to controls. Young people with childhood-onset rheumatic disease is known to have increased risk of CVD and Ciurtin et al. in a review describe some potential ways to improve cardiovascular safety in this population; Mondal et al., instead, describe different aspects of cardiac dysfunction in Juvenile dermatomyositis. As regards therapeutic and drug-related issues, the effects of ketogenic diet on CV outcomes in arthritis are reviewed by Ciaffi et al., reporting a favorable effect specially in RA. Soòs et al. analyzed a possible effect of anti TNF on CV system; they found that anti-TNF treatment may increase ACE and ACE2 in the sera of RA and Ankylosing spondylitis patients and may be associated with disease duration, markers of inflammation and vascular pathophysiology. Toussirot et al. describe the CV risk in spondyloarthritis with highlights on the use of bDMARDs (TNF and probably IL-17i) and specific treatment strategies, as favorable impact on CV risk and disease prognosis.

Sonographic crystal deposits and subclinical inflammation were observed in a study performed by Calabuig et al. in patients with intercritical gout; tophi and a positive Power Doppler signal were linked to carotid atherosclerosis, while the association between hyperuricemia/gout and an increased CVD risk was described by Zhu et al. in a big cohort of patients with long follow up. On the other hand, in a meta-analysis (Zhang et al.) suggests that neither allopurinol nor febuxostat significantly modify—increasing or reducing—the risk of major adverse cardiovascular events in hyperuricemic patients with or without gout.

This special issue aimed to provide an up-to-date state of the art of this constantly evolving picture.

## Author contributions

Both authors listed have made a substantial, direct, and intellectual contribution to the work and approved it for publication.

## Conflict of interest

The authors declare that the research was conducted in the absence of any commercial or financial relationships that could be construed as a potential conflict of interest.

## Publisher's note

All claims expressed in this article are solely those of the authors and do not necessarily represent those of their affiliated organizations, or those of the publisher, the editors and the reviewers. Any product that may be evaluated in this article, or claim that may be made by its manufacturer, is not guaranteed or endorsed by the publisher.
